# Investigation of Gold Nanoparticle Naproxen-Derived Conjugations in Ovarian Cancer

**DOI:** 10.1021/acsmaterialsau.3c00033

**Published:** 2023-06-09

**Authors:** Cansu Umran Tunc, Gizem Kursunluoglu, Munevver Akdeniz, Aybuke Ulku Kutlu, Muhammed Ihsan Han, Mukerrem Betul Yerer, Omer Aydin

**Affiliations:** † Nanothera Lab, Drug Application and Research Center (ERFARMA), Erciyes University, Kayseri 38039, Turkey; ‡ Utah Center for Nanomedicine, University of Utah, Salt Lake City, Utah 84112, United States; § Department of Biomedical Engineering, Erciyes University, Kayseri 38039, Turkey; ∥ Department of Pharmaceutical Chemistry, Erciyes University, Kayseri 38039, Turkey; ⊥ Drug Application and Research Center (ERFARMA), Erciyes University, Kayseri 38039, Turkey; α Auckland Cancer Society Research Centre, University of Auckland, 92019 Auckland, New Zealand; ¶ Department of Pharmacology, Erciyes University, Kayseri 38039, Turkey; ∇ Clinical Engineering Research and Implementation Center (ERKAM), Erciyes University, Kayseri 38040, Turkey; ○ Nanotechnology Research and Application Center (ERNAM), Erciyes University, Kayseri 38040, Turkey

**Keywords:** ovarian cancer, naproxen, drug delivery, gold nanoparticle, thiosemicarbazide, 1,2,4-triazole, drug dispersity

## Abstract

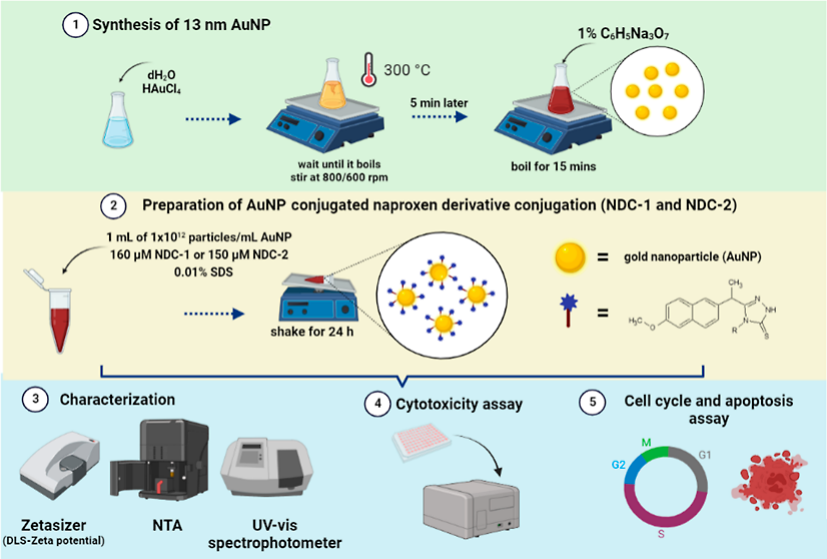

Ovarian cancer, which is one of the most diagnosed cancer types among women, maintains its significance as a global health problem. Several drug candidates have been investigated for the potential treatment of ovarian cancer. Nonsteroidal anti-inflammatory drugs (NSAIDs) demonstrated anti-cancer activity through the inhibition of cyclooxygenase 2 (COX-2) and by inhibiting COX-2-dependent prostaglandin (PG) production. Naproxen is one of the most used NSAIDs and Naproxen-derived compounds (NDCs) may show potential treatment effects on cancer as chemotherapeutic drugs. Although there are successful drug development studies, the lack of solubility of these drug candidates in aqueous media results in limited bioavailability and high variability of patient responses during treatment. Low aqueous solubility is one of the main problems in the pharmaceutical industry in terms of drug development. Nanotechnology-based strategies provide solutions to hydrophobic drug limitations by increasing dispersion and improving internalization. In this study, two different NDCs (NDC-1 and NDC-2) bearing a thiosemicarbazide/1,2,4-triazole moiety were synthesized and tested for chemotherapeutic effects on ovarian cancer cells, which have a high COX-2 expression. To overcome the limited dispersion of these hydrophobic drugs, the drug molecules were conjugated to the surface of 13 nm AuNPs. Conjugation of drugs to AuNPs increased the distribution of drugs in aqueous media, and NDC@AuNP conjugates exhibited excellent colloidal stability for up to 8 weeks. The proposed system demonstrated an increased chemotherapeutic effect than the free drug counterparts with at least 5 times lower IC50 values. NDC@AuNP nanosystems induced higher apoptosis rates, which established a simple and novel way to investigate activity of prospective drugs in drug discovery research.

## 1 Introduction

Cancer is a complex disease caused by multiple factors and treatment is challenging due to the heterogeneity of the cancerous tissue. Chemotherapy is considered as the main treatment modality of cancer followed by radiotherapy and surgery. There has been a tremendous effort for the development of new chemotherapeutics for effective cancer treatment. Ovarian cancer is one of the highly diagnosed cancer types among women with more than 200 thousand deaths per year and has maintained its significance as a global health problem.^1^ There are promising drug candidates that have been investigated for potential treatment of ovarian cancer.^2−4^ However, drug limitations, such as non-selectivity, side effects, low aqueous solubility, and drug resistance, intercept successful therapy.^5^

A high rate of chemotherapy unresponsive cases of ovarian cancer exhibits increased expression levels of cyclooxygenase-2 enzyme (COX-2), suggesting that COX-2 involves pathways associated with chemotherapy sensitivity of this cancer type.^6^ Non-steroidal anti-inflammatory drugs (NSAIDs) are known to repress prostaglandin (PG) production, and their activity as a COX-2 inhibitor was demonstrated in clinical studies.^7^ Naproxen, a well-known NSAID, and Naproxen-derived compounds (NDCs) recently have been investigated for their anticancer activities^8−14^

Our group has pioneered the synthesis and usage of the (S)-Naproxen thiosemicarbazide/1,2,4-triazol derivatives as potential anticancer compounds.^15,16^ Molecules containing a thiosemicarbazide structure have been the focus of researchers in drug discovery due to their broad spectrum of pharmacological activities. Most promisingly, they have anticancer effects in a wide range of tumor types.^8,17−21^ Our previous research showed that (S)-Naproxen thiosemicarbazide/1,2,4-triazol compounds inhibited breast cancer progression in vitro and in vivo.^15^ Although the tested compounds exhibited a high cancer therapy effect through cytotoxicity, apoptosis, and Bcl-2 inhibition, most of these compounds also suffered from low aqueous solubility, which is one of the most important factors that prevents the demonstration of the real potential of new drug candidates due to limited bioavailability.

Nanoparticle-based drug delivery systems can be the answer to many of the limitations of chemotherapy with the ability to disperse hydrophobic drugs in water and selective distribution through the tumor tissue due to enhanced permeability and retention (EPR) effects. Gold nanoparticles (AuNPs), one important class of nanosized materials, have been utilized for many biomedical applications, including but not limited to sensing, bioimaging, gene delivery, and drug delivery.^22,23^ Fundamental properties of AuNPs such as low cytotoxicity, minimal immune response, high surface area, tunable size, easy modification, and high cellular internalization make them excellent vehicles for nanomedicine applications, especially for drug delivery.^25^

In this study, two Naproxen 1,2,4-triazol-derived compounds (NDC-1 and NDC-2), from our previously published research^15^ were investigated as drugs for treatment of ovarian cancer by attaching these hydrophobic compounds to AuNPs for high aqueous dispersion. Here, we described the construction of NDC-1@AuNP and NDC-2@AuNP nanoconjugates as potential chemotherapeutics against ovarian cancer by direct binding of drug molecules on the surface of AuNPs. It was determined that the drug molecules were successfully conjugated to AuNPs and perfectly dispersed in water. Dynamic light scattering (DLS) and nanoparticle tracking analysis (NTA) evaluations showed that the carrier system maintained its physical properties with excellent colloidal stability for at least 8 weeks at room temperature. Cytotoxicity and the apoptosis effect of the compounds on ovarian cancer were further investigated. Our proposed system demonstrated an increased chemotherapeutic effect than the free compound counterparts with at least 5 times lower IC50 values and higher apoptosis rates, which established a simple and novel way to investigate the activity of prospective drugs in drug discovery research. To the best of our knowledge, this is the first study testing Naproxen derivatives on ovarian cancer as well as using AuNP-based dispersion and delivery for enhanced treatment.

## 2 Methods

### 2.1 Synthesis of the Compounds

Compounds, NDC-1 and NDC-2, were synthesized according to the previously published method.^15^ Briefly, the solution of thiosemicarbazide (Cat. no. T33405, Sigma-Aldrich Co., St Louis, MO, USA), compounds (0.01 mol) were heated in a 4 N sodium hydroxide solution (20 mL) under Radley (Heidolph Plug MR Radley) at 120 °C for 24 h. The solution was set to pH 6 by using glacial acetic acid after cooling at room temperature. The products were precipitated, filtered, and washed with dH_2_O. The compounds were obtained by recrystallization from ethanol. Characterization results of the synthesized compounds have been shown in our previous study.^15^

### 2.2 Synthesis of AuNPs

AuNPs were synthesized using the citrate reduction method.^23^ Briefly, 40 mg of gold chloride salt (HAuCl_4_.3H_2_O) (Cat. no. 520918-5G, Sigma-Aldrich Co., St Louis, MO, USA) was dissolved in 100 mL of ultrapure dH_2_O, and the solution was heated until boiling on a magnetic stirrer. While boiling, a 10 mL of 38.8 mM sodium citrate (Na_3_C_6_H_5_O_7_) (Cat. no. W302600-1KG-K, Sigma-Aldrich Co., St Louis, MO, USA) solution, which induces reduction of gold salt, was rapidly added into the boiling solution. The solution was kept boiling for 15 min and left at room temperature for cooling.

### 2.3 Preparation of NDC-1@AuNPs and NDC-2@AuNPs

1 mL of 1 × 10^12^ particles/mL AuNPs were mixed with NDC-1 and NDC-2 compounds at final concentrations of 160 and 150 μM, respectively. Sodium dodecyl sulfate (SDS) was added to the solution to prevent instant aggregation in a final 0.01% SDS. The mixture was left shaking for 24 h at room temperature for the attachment of compounds to the surface of AuNPs through SH/Au bonding. The NDCs@AuNPs were centrifuged at 13,000 rpm for 20 min and the concentration of the NDCs were determined using UV/Vis spectroscopy.

### 2.4 Stability Assays

DLS (Malvern, Nano ZS, UK) and UV/vis spectroscopy (Shimadzu Corp., UV-2700) were used for both characterization of the drug-AuNP conjugates and stability assessment. After drug conjugating, hydrodynamic size and absorbance spectra of the bare AuNPs and NDCs@AuNPs were determined. The synthesized AuNPs and NDCs@AuNPs were diluted in dH_2_O to a final AuNP concentration of 2 × 10^11^ particles/mL and DLS and UV/vis spectroscopy measurements were taken. For the stability assessment, the prepared NDC@AuNP conjugates were incubated at RT for 8 weeks and Zeta-sizer (Malvern, Nano ZS, UK) for zeta potential measurements and hydrodynamic diameter distribution (DLS), UV/vis spectroscopy, and nanoparticle tracking analysis (NTA) (Malvern, Nanosight NS300, UK), were utilized each week for stability.

### 2.5 Cell Culture

OVCAR3, ovarian cancer cells were cultured in a RPMI (Cat. no. 21875-034, Gibco, UK) medium containing 10% FBS and supplemented with 100 units/mL penicillin/streptomycin. The cultures were incubated at 37 °C in a humidified atmosphere with 5% CO_2_. Cellular morphological changes were characterized under a light microscope (Zeiss, Axio Vert A1, Germany).

### 2.6 Cytotoxicity Assay

IC50 values of NDC-1, NDC-2, NDC-1@AuNPs, and NDC-2@AuNPs were determined on OVCAR3 ovarian cancer cells. 10,000 cells were seeded in 96 wells and left for attachment. The cells were treated with the drugs and incubated for 48 h. The control cells were treated with 0.01% SDS solution. The viability of cells was determined using a resazurin assay.^100^ The viability of cells was normalized to the control. IC50 values were calculated using GraphPad Prism 8 software.

### 2.7 Cell Cycle and Apoptosis Assay

The cell cycle phase distributions and apoptotic population of OVCAR3 cells were determined using flow cytometry. The cells were seeded in 24 wells at 40,000 cells/well and treated with NDC-1 and NDC-1@AuNPs at 16.06 μM drug concentration and NDC-2 and NDC-2@AuNPs at 17.63 μM drug concentration for 48 h. Following the drug incubations, the cells were collected and fixed using 70% ethanol at 4 °C overnight for the cell cycle assay and directly stained with Annexin V-FITC and PI for apoptosis analysis using an apoptosis assay kit (Invitrogen) by following our published protocol.^101^ For cell cycle analysis, the fixed cells were incubated with RNase A solution for 30 min at 37 °C and stained with PI. The cells were analyzed using a Guava EasyCyte flow cytometer (Millipore, USA) and the phase distributions were determined according to the DNA content.

## 3 Results and Discussion

### 3.1 SH Containing Hydrophobic Compounds Were Successfully Conjugated to AuNPs

**Figure 1:**
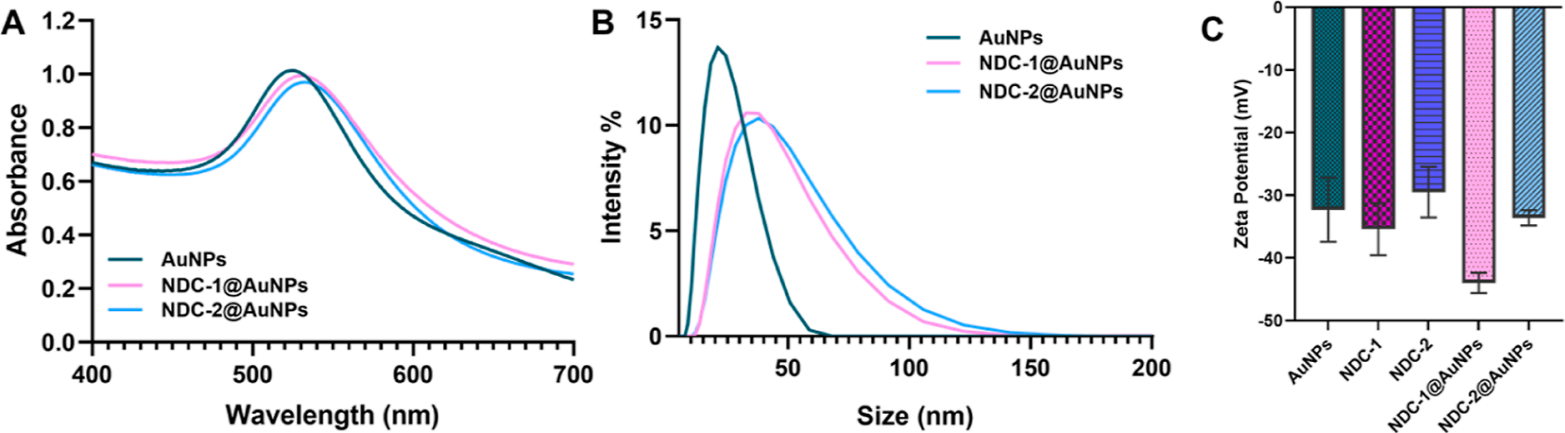
(A) UV/vis spectra, (B) hydrodynamic size, and (C) Zeta potential of AuNPs, NDC-1@AuNPs, and NDC-2@AuNPs; drug conjugating caused size increase and resulted in shifts on UV/vis spectra and DLS results of AuNPs.

AuNPs have various properties that increase their use in nanomedicine, the most important point is that they can be conjugated with different ligands, oligonucleotides, drugs, and biomarkers on the AuNP surface. Because the Au surface is suitable for thiol functionalization chemistry. Thus, they can be conjugated with drugs containing various thiol structures. The properties of AuNPs included rapid and simple synthesis, safety, stability, biocompatibility, and low cytotoxicity making them especially worthy for cancer treatment and drug delivery.^24,25^ In this study, we primarily aimed to provide a hydrophobic drug conjugation by utilizing the drug delivery capability of gold nanoparticles. Two Naproxen 1,2,4-triazol-derived compounds (NDC-1 and NDC-2) were used for conjugation on AuNPs. The chemical structure of NDCs, including NDC-1 and NDC-2, is shown in Figure S1. The loading concentrations were approximately 0.15 mg per mg Au for both NDC-1 and NDC-2. The plasmonic features of AuNPs allow following the drug loading with spectroscopic techniques as well as eye monitoring. Excess amounts of compounds caused aggregation and color change in AuNPs due to the biosensor properties of AuNPs, which allowed us to optimize the loading with a maximum efficiency. To further characterize NDCs@AuNPs, UV/vis spectroscopy analysis and surface charge characterizations were performed. UV/vis spectra, hydrodynamic size, and surface charge analysis of bare AuNPs and NDCs@AuNPs are shown in Figure 1. The spectrum of AuNPs depends on the size of the nanoparticles. A maximum absorbance value of 13 nm AuNPs was 520 nm, and the absorbance values of both NDC-1@AuNPs and NDC-2@AuNPs were shifted to 530 nm, indicating the successful conjugation of the drugs on the surface. The average size of bare AuNPs was 13 nm, while NDCs@AuNPs was approximately 35 nm. Zeta potentials of the conjugates were generally between −35 and −40 mV.

Drug-conjugated AuNPs made with many thiol-terminated drug molecules, usually decorated with a thiol-Au covalent bond on their surface, are highly effective nanotechnological drugs.^26^ To form novel NDC-1 and NDC-2 drug conjugate gold nanoparticles, thiol-Au covalent bonds were used and then their solubility, stability, and anti-cancer efficacy were evaluated. Previously, AuNPs have been effectively used for drug dispersity in the literature.^27−31^ Zhang et al. attached Paclitaxel to AuNPs via DNA linkers for increased solubility and effectiveness.^30^ In another study, Paclitaxel was attached to AuNPs through thiol-terminated polyethylene glycol molecules^32^ and enhanced performance was obtained due to higher solubility and stability.^31^ Even though the studies show that hydropic drug attachment to AuNPs improved the drug effectiveness, dependence on the usage of linkers makes the system complex and expensive. In addition, macromolecules, such as DNA and PEG, limit the number of the attached drug molecules on AuNPs, considering the overall surface area of the nanoparticles. On the other hand, thiosemicarbazide structures have an affinity to metals and the thiosemicarbazide-bearing compounds directly attached to the surface of AuNPs through thiol-Au bonding. This strategy makes the nanoconjugation simple and cost effective and single NPs can bear a high number of small drug molecules with minimal steric blocking.

### 3.2 Drug-Conjugated AuNPs Provided Well Dispersity to the Drugs

Due to optical properties of AuNPs, spectra of these nanoparticles depend on their size and aggregation.^33,34^ UV/vis spectroscopy allows fast and easy determination of size of AuNPs. In our study, whether NDC-1 and NDC-2 were conjugated to the AuNPs surface were determined from the shift in λ_max_. Thus, the first-step characterization was performed by using UV/vis. UV/vis spectra of AuNP alone and AuNP conjugated with different NDCs, and it was observed that the λ_max_ value shifted from 520 nm to 530 nm after conjugation. Because we determined the shift in wavelength, our data suggested that NDC@AuNP drug conjugation was successful. DLS is an analytical technique used for the determination of hydrodynamic size of nanoparticles in aqueous media. DLS is highly convenient for the determination of hydrodynamic size of AuNPs and its conjugates.^35^ Thus, we further analyzed our nanoconjugates with DLS.

NDC-1 and NDC-2 were dissolved in DMSO and when the same number of conjugated drugs were added into aqueous media their suspension presented a blurry appearance indicating the poor dispersity. Hydrophobic drugs cause large particles in water. Hydrodynamic sizes of bare and AuNP-conjugated drugs are shown in Figure S2. The sizes of free NDC-1 and NDC-2 were approximately 255 nm and 150 nm, respectively, with very wide distributions reaching up to 600 nm. When the drugs were conjugated with 13 nm AuNPs, the hydrodynamic sizes of NDC-1@AuNPs and NDC-2@AuNPs were approximately 40 nm, respectively, and large particles disappeared completely, which indicates well distribution. Although free hydrophobic drug molecules formed large particles in aqueous media, NDC-1@AuNPs and NDC-2@AuNPs showed a well hydrodynamic distribution. The results indicated that the NDC-1 and NDC-2 compounds were successfully attached to the surface of the AuNPs with high efficiency.

### 3.3 Drug-AuNP Conjugates Demonstrated Excellent Stability

**Figure 2:**
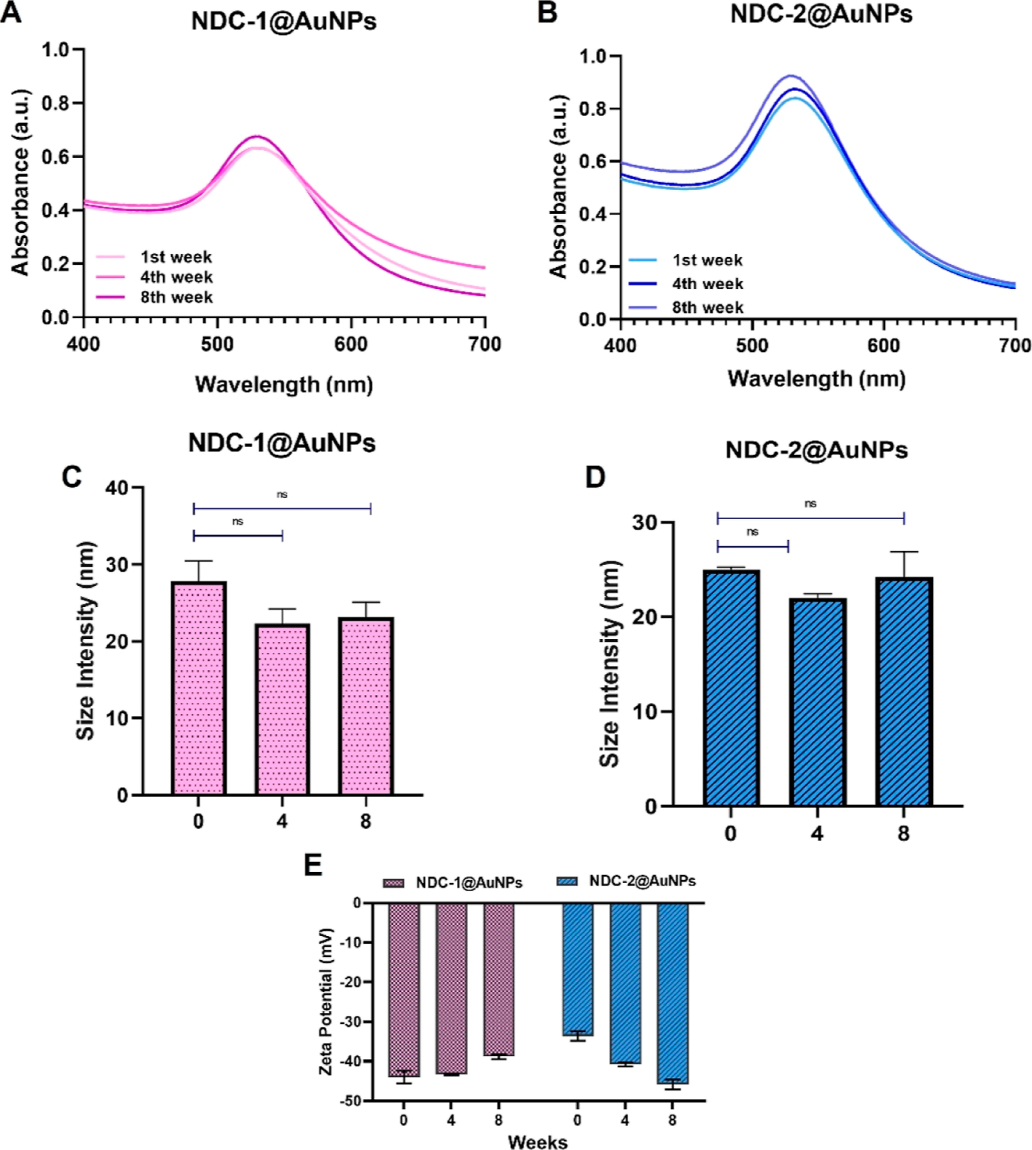
Stability evaluation of NDC-1@AuNPs and NDC-2@AuNPs. UV/vis spectra of (A) NDC-1@AuNPs and (B) NDC-2@AuNPs, hydrodynamic size of (C) NDC-1@AuNPs, (D) NDC-2@AuNPs, (E) zeta potential of NDC-1@AuNPs, and NDC-2@AuNPs.

Maintaining stability of drug-conjugated nanoparticles over time is crucial for efficacy, safety, and long shelf-life.^36^ The stability of drug-conjugated nanoparticles is assessed based on parameters, such as hydrodynamic radius, zeta potential, and concentration.^37^ Here, stabilities of the NDC-1@AuNP and NDC-2@AuNP conjugates were assessed by NTA, UV/vis spectroscopy, DLS, and zeta potential measurements after incubating at room temperature at 0th, 4th, and 8th weeks. The size, UV/vis spectroscopy, and zeta potential results showed that NDCs-AuNPs maintained their physical properties for 8 weeks (Figure 2). DLS was used to observe changes in the hydrodynamic diameter of the NDC-1@AuNPs and NDC-2@AuNPs over time at room temperature. NDC-1@AuNPs and NDC-2@AuNPs conjugates showed improved stability with mean diameters of 24.4 and 23.7 nm, respectively. No indication of size increase or spectrum shift was detected in UV/vis spectroscopy analysis between weeks 1 and 8 (Figure S3). The maximum absorbance value of NDC-1@AuNPs and NDC-2@AuNPs was 530 nm immediately after preparation and there was no increase during the 8 weeks of period. In addition, the zeta potential of the nanodrug conjugates remained without significant changes. According to our results, size increase and absorbance shift of the conjugates were not observed, indicating the remaining colloidal stability of the conjugates. Zeta potential is another parameter mostly used to indicate stability. Zeta potential of the conjugates was generally between −35 and −40 mV and did not change extremely over time. The values provided sufficient surface charge to prevent the aggregation of nanoparticles. These results are consistent with the DLS, NTA, and UV/vis spectroscopy results, which did not show any indication of aggregation. AuNPs have high van der Waals forces, which makes NPs susceptible to aggregation.^38^ Following the synthesis by the citrate reduction method, AuNPs can remain in a colloidal suspension only due to the electrostatic repulsion derived from the negatively charged citrate ions on the NP surface. Any impurity can cause the removal of citrate ions from the surface, giving even higher van der Waals forces to AuNPs and causing aggregation, which is irreversible almost every time. After binding the NDCs to the surface, a new electrostatic repulsion equilibrium was reached due to the negatively charged compounds covering the surface.

Another important parameter for stability is the NP concentration. Aggregation of NPs decreases the number of NP per mL in colloidal suspension at concentration analysis. Changes in the concentration of the conjugates were investigated by using NTA. It was observed that the conjugates had a particle concentration more than 1 × 10^11^ particles/mL for 8 weeks. As shown in Table 1, the concentration of the NPs was followed using NTA analysis and it was determined that the concentration of NDC-1@AuNPs and NDC-2@AuNPs was approximately 1 × 10^11^ particles/mL after synthesis and conjugation of the drugs. As can be seen from Table 1, the size of both NDC-1@AuNPs and NDC-2@AuNPs remained constant over the 8 week period. Whereas, the particle concentration decreased over the same time period. On the other hand, no precipitation was detected in both conjugations, both visually and as a result of the maximum UV/vis absorbance. Therefore, no increase in particle size or change in formulation stability was observed. As a result, the nanoconjugates we developed both preserved their properties and remained stable at room temperature for 8 weeks. Further, our stability results showed that AuNPs maintained their colloidal stability and showed no indication of aggregation, which was the result of successful attachment of the NDCs and proved the stable conjugation.

**Table 1:** Stability of NDC-1@AuNPs and NDC-2@AuNPs for 0th, 4th, and 8th Weeks Including Hydrodynamic Size and Concentration (Particle/mL)

	NDC-1@AuNPs	NDC-2@AuNPs		
weeks	size (nm)	concentration (NP/mL)	size (nm)	concentration (NP/mL)
0	27.8 ± 2.65	3.65 ± 0.92 × 10^11^	25.0 ± 0.25	6.45 ± 0.06 × 10^11^
4	22.3 ± 1.89	2.9 ± 0.7 × 10^11^	22.0 ± 0.44	3.62 ± 0.8 × 10^11^
8	23.1 ± 2.0	1.62 ± 0.2 × 10^11^	24.2 ± 2.7	1.84 ± 0.07 × 10^11^

### 3.4 Enhanced Solubility of Drugs via AuNPs Increased Cytotoxicity

**Figure 3:**
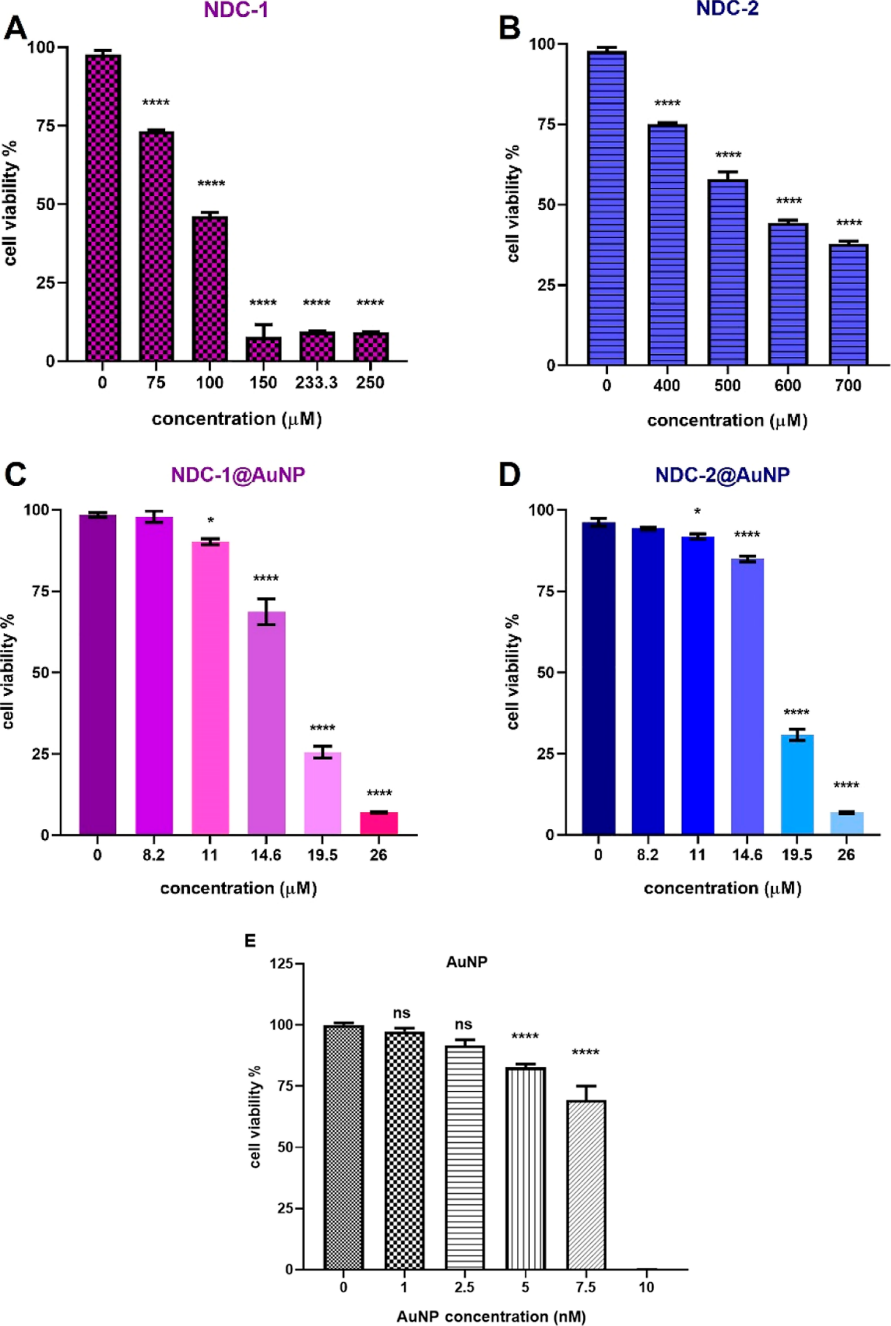
Cytotoxicity of (A) NDC-1, (B) NDC-2, (C) NDC-1@AuNPs, and (D) NDC-2@AuNPs, and (E) bare AuNPs at various concentrations in OVCAR cells. The experiments were performed with a minimum of three replicates, and values represent the mean ± standard deviation of these repetitions. *p* value represents *, *p* < 0.05; ****p < 0,0001.

The main problem with chemotherapeutic drugs is that they are often very hydrophobic. For this reason, these water-insoluble drugs are dissolved in different chemical solvents or used in high concentrations to increase their effectiveness and bioavailability. The main purpose of drug delivery with nanoparticles is to reduce drug toxicity and to enhance the therapeutic activity with less drug concentration and dosage.^39^ Insolubility of drug molecules causes the formation of large particles in aqueous media, which leads to poor internalization by cells. Increasing the distribution of drugs is essential for cellular uptake and bioavailability. Thus, when the drug molecules were dispersed using AuNPs, their cytotoxic effect was increased. In this study, AuNPs were used to transport Naproxen-derived chemotherapeutics due to their large surface area. By ensuring the cellular uptake of hydrophobic drug molecules with AuNPs, organic/inorganic solvent-induced toxicity will be eliminated. IC50 values of NDC-1 and NDC-2 compounds were determined on OVCAR3 cells at 48 h incubation. The cells were treated with NDCs@AuNPs, increasing NDC concentrations between 8.2 and 26 μM containing AuNPs at minimum 0.87 nM and maximum 2.76 nM concentrations. At this concentration range, the AuNPs were nontoxic to the cells. Even though the AuNP treatments at high concentrations showed a toxic effect on cells, one might note that gold has an inert nature and as the NPs were reduced with sodium citrate, the increasing concentration of citrate ions in the colloidal suspension decreased the cell viability. Removing the citrate from the AuNP suspension causes immediate aggregation of NPs. Thus, the bare AuNP treatments were performed in the presence of citrate ions. However, covering the surface with NDCs prevented aggregation, which allowed removal of citrate ions via centrifugation. Therefore, there was contribution to the toxic effect of the NDCs from neither AuNPs nor citrate ions. The IC50 values of NDC-1 and NDC-2 were 91.96 and 437.80 μM in their free forms, respectively. While the IC50 values decreased to 16.06 and 17.63 μM for NDC-1@AuNPs and NDC-2@AuNPs, respectively (Figure 3). These results showed that increased solubility via AuNPs enhanced the cytotoxic effect of the NDC-1 and NDC-2 compounds on cancer cells.

The preparation of water-insoluble drugs as nanoformulations causes an increase in the surface area and thus, an increase in solubility. The safe development of nanotechnology and the use of NPs require the consideration of cellular toxicity of those NPs.^40^ Seko et al. develop and characterize polylactide-co-glycolide (PLGA) nanoparticles (NPs). They determined their effects on the line IC50 values. Docetaxel solution was found to be more toxic than docetaxel-loaded PLGA NPs.^41^ Again, many studies have been carried out in the literature to examine the cytotoxicity effect of doxorubicin and methotrexate (two of the most important chemotherapeutic drugs). In these studies, where only drug and drug/NPs IC50 values were compared, significant differences were observed between the two groups. Compared to DOX only, DOX/Bromelain AuNPs reduced the IC50 by 2.5 times.^42^ These results and the results we obtained in our study show that the toxic effect can be decreased by using nanoparticles.

### 3.5 Nanoformulization Provided Enhanced Apoptosis

**Figure 4:**
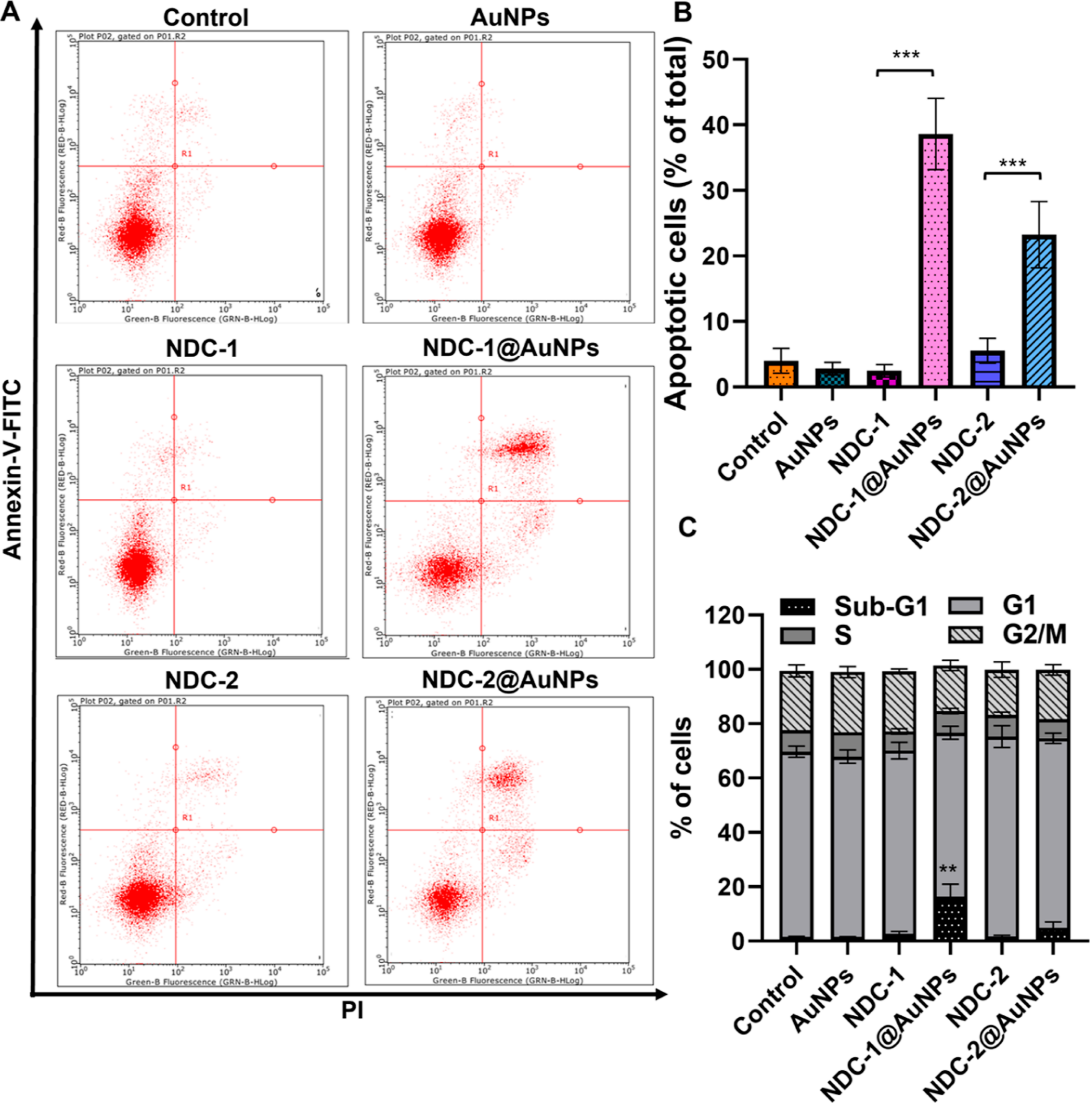
(A) Flow cytometric analysis of OVCAR3 cell apoptosis, (B) total apoptosis rates of cancer cells treated with free and nano-conjugated compounds, and (C) cell cycle phase distributions of OVCAR3 cells after treatment with NDCs and NDCs@AuNPs. *p* value represents **, *p* < 0.01, ***, *p* < 0.001.

Apoptosis is one of the most preserved mechanisms in cellular machinery. It provides a clearance of damaged or mutated cells. Induction of apoptosis in cells is essential for cancer treatment. Thus, we further investigated the ability of the synthesized compounds for the induction of apoptosis in ovarian cancer cells. Following the cytotoxicity assessment of the NDCs and NDCs@AuNPs, apoptosis rates of the ovarian cancer cells were determined. The apoptotic effect of NDC-1@AuNPs and NDC-2@AuNPs was also confirmed with cell cycle phase distributions of the ovarian cancer cells. The cells were treated with NDC-1 and NDC-1@AuNPs at 16.06 μM, while NDC-2 and NDC-2@AuNPs at 17.63 μM concentrations. The results showed that NDC-1@AuNPs and NDC-2@AuNPs induced approximately 40% and 25% apoptosis, respectively (Figure 4). On the other hand, NDC-1 and NDC-2 alone did not cause significant apoptosis on cancer cells at the tested concentrations. At increased concentrations, the NDCs formed crystal constructions due to high hydrophobicity. These large size structures caused the disruption of cell membrane rather than inducing apoptosis, which led to an insufficient number of cells for further analysis.

Cell cycle phase distribution analysis confirmed the enhanced apoptosis with increased Sub-G1 ratio (approximately 15%) on ovarian cancer cells at NDC-1@AuNP treatment. Our results showed that NDCs@AuNPs caused significantly high rates of apoptosis, while free NDCs showed no apoptosis effect. Complementary to the literature, the apoptotic rate of the cancer cells demonstrated that the hydrophobic compounds’ solubility increased when attached to AuNPs leading to enhanced drug internalization.

## 4 Conclusions

Several chemotherapeutic candidates have been tested for effective treatment in research and only a few manage to enter the clinical trial stages. The majority of the failure of the new drugs has been due to a lack of aqueous solubility. Poor solubility of the molecules causes serious problems, such as decreased bioavailability and restricted cellular internalization. Even a number of successful marketed drugs have been reformulated using drug delivery technologies to enhance safety and efficacy. Particularly, nanotechnology-based carrier systems offer a much more effective solution to these problems than conventional carrier systems, thanks to their large surface areas, high carrying capacities, and their ability to carry both hydrophilic and hydrophobic agents. The use of nanocarrier systems is seen as an effective strategy to increase the bioavailability and therapeutic efficacy of drugs with low solubility, especially in aqueous media. Here, we investigated two NDCs as potent chemotherapeutics in nanoformulation for the treatment of ovarian cancer in vitro. AuNPs were utilized to transport NDCs. The molecules showed toxic effects at high concentrations, while NDCs@AuNPs showed cytotoxicity and apoptosis effects at lower concentrations by providing enhanced dispersity of the compounds in aqueous media and increasing the availability for cellular internalization. AuNPs provide an inert carrier system and enable effective loading with a high surface area, resulting with a high drug per mass carrier. By ensuring the cellular uptake of hydrophobic drug molecules with AuNPs, organic/inorganic solvent-induced toxicity may be eliminated. This simple and one-step AuNP-based hydrophobic drug dispersity can be an answer to the unmet clinical need.

## Supporting Information Available

Chemical structure of the compounds, additional size distribution graphs, and UV/vis spectra of NDS@AuNP conjugates (PDF)
