# Supplementary material for: Investigation of Gold Nanoparticle Naproxen-Derived Conjugations in Ovarian Cancer
Source: ACS Mater Au. 2023 Jun 9;3(5):483–91. doi: 10.1021/acsmaterialsau.3c00033 (PMC10510500; doi:10.1021/acsmaterialsau.3c00033)
Supplement: Supplementary file 1 — mg3c00033_si_001.pdf [file mg3c00033_si_001.pdf]

Supporting Information

Investigation of Gold Nanoparticle Naproxen Derived Conjugations in Ovarian Cancer

Cansu Umran Tunc[1,2,3#], Gizem Kursunluoglu[1#], Munevver Akdeniz[1,3], Aybuke Ulku Kutlu[1,3], Muhammed Ihsan Han[4,5,6], Mukerrem Betul Yerer[5,7], Omer Aydin[1,3,8,9*]

[1]Nanothera Lab, Drug Application and Research Center (ERFARMA), Erciyes University, 38039 Kayseri, Turkey.

[2]Utah Center for Nanomedicine, University of Utah, Salt Lake City, UT, 84112, USA

[3]Department of Biomedical Engineering, Erciyes University, 38039, Kayseri, Turkey.

[4]Department of Pharmaceutical Chemistry, Erciyes University, 38039, Kayseri, Turkey.

[5]Drug Application and Research Center (ERFARMA), Erciyes University, 38039, Kayseri, Turkey.

[6]Auckland Cancer Society Research Centre, University of Auckland, 92019, Auckland, New Zealand

[7]Department of Pharmacology, Erciyes University, 38039, Kayseri, Turkey.

[8]Clinical Engineering Research and Implementation Center (ERKAM), Erciyes University, 38040, Kayseri, Turkey.

[9]Nanotechnology Research and Application Center (ERNAM), Erciyes University, 38040, Kayseri, Turkey.

#These authors contributed equally to this work.

*** Corresponding Author**

Assistant Prof Omer Aydin

Department of Biomedical Engineering

Erciyes University, Kayseri/TURKEY, 38039

Email: biomer@umich.edu , omeraydin@erciyes.edu.tr

Phone: +90-352-207-6666 X 32984

Supporting information: Chemical structure of the compounds, additional size distribution graphs and UV/Vis spectra of NDS@AuNPs conjugates

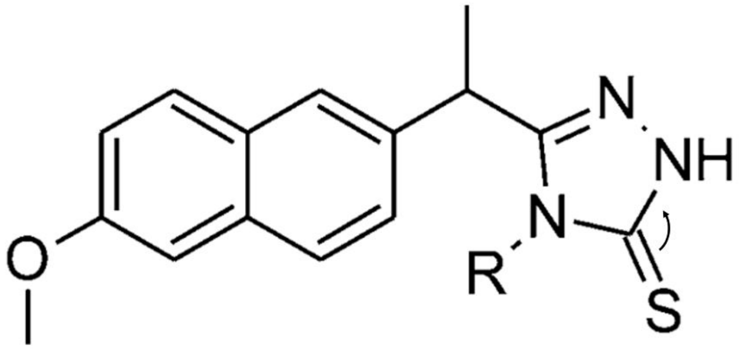

Figure S1. Chemical structure of naproxen derivatives compound (NDC). The triazole structure has tautomeric form in solution yielding an SH group.

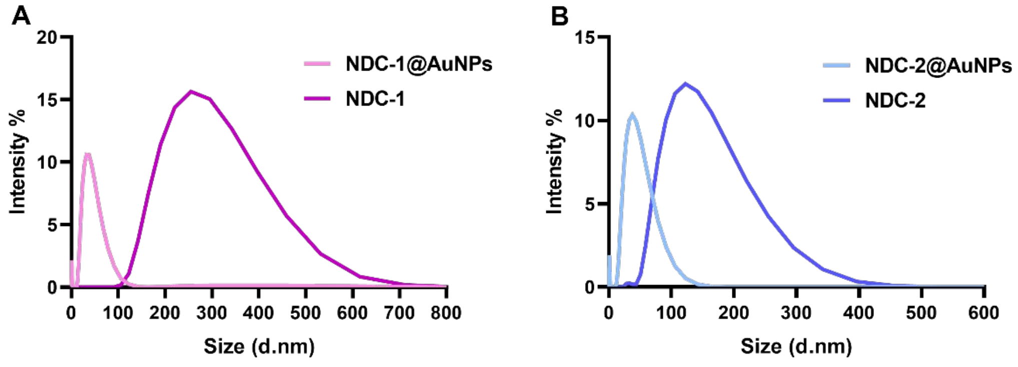

Figure S2. Comparison of particle size distributions of **(A)** NDC-1@AuNPs and NDC-1, **(B)** NDC-2@AuNPs and NDC-2. Although free hydrophobic drug molecules formed large particles in aqueous media, NCD-1@AuNPs and NCD-2@AuNPs showed well hydrodynamic distribution.

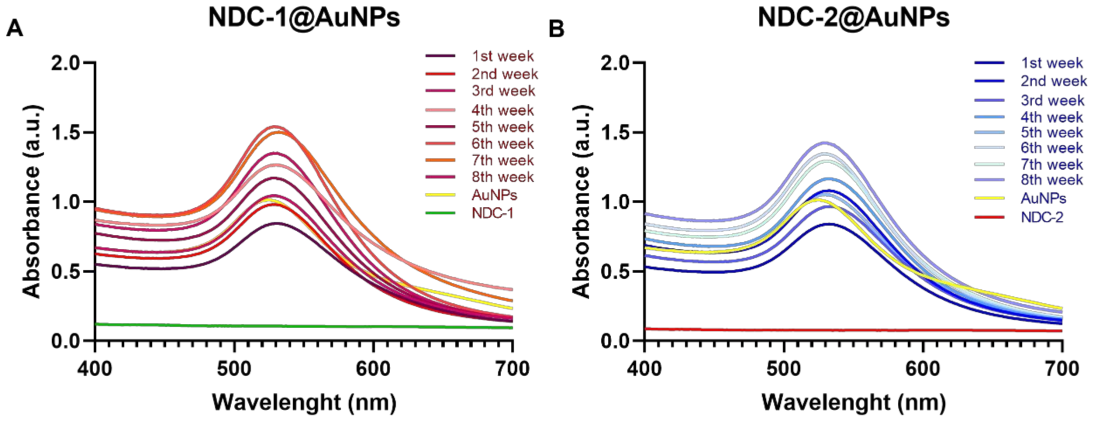

Figure S3. UV/Vis spectra of **(A)** NDC-1@AuNPs and **(B)** NDC-2@AuNPs for stability evaluation at 8 weeks.
